# Novel Inactivated Subtype B Avian Metapneumovirus Vaccine Induced Humoral and Cellular Immune Responses

**DOI:** 10.3390/vaccines8040762

**Published:** 2020-12-14

**Authors:** Yuanling Bao, Mengmeng Yu, Peng Liu, Fujun Hou, Farooque Muhammad, Zhihao Wang, Xinyi Li, Zhuo Zhang, Suyan Wang, Yuntong Chen, Hongyu Cui, Aijing Liu, Xiaole Qi, Qing Pan, Yanping Zhang, Li Gao, Kai Li, Changjun Liu, Xijun He, Xiaomei Wang, Yulong Gao

**Affiliations:** 1 State Key Laboratory of Veterinary Biotechnology, Avian Immunosuppressive Diseases Division, Harbin Veterinary Research Institute, The Chinese Academy of Agricultural Sciences, Harbin 150069, China; baoyuanling0816@163.com (Y.B.); yumengmeng1029@126.com (M.Y.); lxpliupeng@126.com (P.L.); farooqlaghari1986@gmail.com (F.M.); a17854265231@163.com (Z.W.); 18728192312@163.com (X.L.); zhangzhuo02@caas.cn (Z.Z.); 15153832031@163.com (S.W.); chenyt2015@163.com (Y.C.); cuihongyu@caas.cn (H.C.); liuaijing@caas.cn (A.L.); qixiaole@caas.cn (X.Q.); panqing@caas.cn (Q.P.); zhangyanping03@caas.cn (Y.Z.); gaoli@caas.cn (L.G.); likai01@caas.cn (K.L.); liuchangjun@caas.cn (C.L.); hexijun@caas.cn (X.H.); wangxiaomei@caas.cn (X.W.); 2 Aohan County Breeding and Extension Center, Chifeng, Inner Mongolia 024300, China; aohhfj@163.com

**Keywords:** avian metapneumovirus, inactivated vaccine, adjuvant

## Abstract

Avian metapneumovirus (aMPV), a highly contagious agent, is widespread and causes acute upper respiratory tract disease in chickens and turkeys. However, currently, there is no vaccine licensed in China. Herein, we describe the development of an inactivated aMPV/B vaccine using the aMPV/B strain LN16. Combined with a novel adjuvant containing immune-stimulating complexes (ISCOMs), the novel vaccine could induce high virus-specific and VN antibodies. In addition, it activated B and T lymphocytes and promoted the expression of IL-4 and IFN-γ. Importantly, boosting vaccination with the inactivated aMPV/B vaccine could provide 100% protection against aMPV/B infection with reduced virus shedding and turbinate inflammation. The protection efficacy could last for at least 6 months. This study yielded a novel inactivated aMPV/B vaccine that could serve as the first vaccine candidate in China, thus contributing to the control of aMPV/B and promoting the development of the poultry industry.

## 1. Introduction

The avian metapneumovirus (aMPV) is a member of the Paramyxoviridae family. Since its first detection in South Africa in 1978, aMPV has a global distribution in poultry-producing regions, except for Oceania [1]. Presently, it is among the main pathogens causing respiratory tract infections and egg production decline in poultry-breeding countries [2]. Based on the nucleotide sequence of the attachment (G) protein gene, aMPV has been classified into four subgroups (A, B, C, and D). Particularly, subtypes A (aMPV/A) and B (aMPV/B) are the most prevalent and have been reported worldwide, except in the United States, Canada, and Australia, whereas the main epidemic areas for subtypes C (aMPV/C) and D (aMPV/D) are the United States and France, respectively [3,4].

aMPV is the causative agent of turkey rhinotracheitis in turkey flocks and swollen head syndrome in chickens. It primarily results in respiratory disease characterized by coughing, clear to turbid nasal discharge, foamy conjunctival secretions, and infra-orbital swelling [5,6]. In laying poultry, a transient drop in egg production has also been observed [7]. Morbidity rate can be as high as 100%, and mortality rate varies from 5 to 10% with a single aMPV infection. Moreover, field studies have found that chickens are more likely to develop viral or bacterial infections following aMPV infection, thus increasing disease severity and mortality rate. The secondary infection might be related to the immunosuppression effect caused by the virus [8,9,10]. Secondary agents, including *Bordetella avium*, Pasteurella-like organisms, *Mycoplasma gallisepticum*, *Chlamydophila*, and *Ornithobacterium rhinotracheale* have all been shown to exacerbate and prolong the clinical disease [11].

Particularly, in China, serological studies have shown that aMPV infection is common in breeding flocks with a 100% positive rate in multiple breeding farms [12]. In addition, aMPVs subtypes A, B, and C have all been reported in poultry farms [12]. Among these three subtypes, aMPV/B is the main subtype and affects the layer and broiler breeding industry [13]. To date, aMPV/B and aMPV/C have been successfully isolated through chicken embryo culture and Vero or CEF cell culture. In 2016, the aMPV/B LN16 strain was isolated from breeders, and its pathogenicity to chickens has been investigated [14]. Meanwhile, aMPV/C has been isolated from Muscovy ducks in 2014 [15]. In addition, it can replicate in wild birds, and wild migratory birds have been implicated in the spread of the disease [16]. Although vaccination strategies are currently the main means of control, there is no licensed aMPV vaccine available in China. Therefore, the continuous spread of aMPV poses a serious threat to the development of the poultry industry and generates huge economic losses.

In this study, we developed an inactivated aMPV/B vaccine using the aMPV/B strain LN16, which was successfully isolated from chickens. We further demonstrated that the inactivated vaccine combined with novel adjuvant containing immune-stimulating complexes (ISCOMs; herein aMPV/B-novel) can induce effective humoral and cellular immunity. Boosting immunization of the activated aMPV/B vaccine provided 100% protection without apparent turbinate inflammation after challenge, and protective immunity could last for at least 6 months. Thus, our findings provide a novel aMPV/B vaccine candidate with great potential to address the continuous spread of aMPV and the threats it poses.

## 2. Methods

### 2.1. Experimental Animals

One-day-old specific-pathogen-free (SPF) chickens were obtained from Harbin Weike Biotechnology Co., Ltd (Harbin, China). They were housed under negative pressure at 25–33 °C and received feed and water ad libitum for 3 weeks.

### 2.2. Ethical and Regulatory Approval

All animal studies with chickens were approved by the Review Board of Harbin Veterinary Research Institute, Chinese Academy of Agricultural Sciences (HVRI-IACUC-2020-008). All animal procedures were performed according to international standards on animal welfare.

### 2.3. Cell and Virus

Vero E6 cells were grown in Dulbecco’s modified Eagle’s minimal essential medium (DMEM; Sigma, Shanghai, China) supplemented with 10% fetal bovine serum (FBS; Sigma, Shanghai, China) at 37 °C in 5% CO_2_. The aMPV/B LN16 strain was propagated in monolayers of Vero E6 cells grown in DMEM with 1% FBS and 1% antibiotics. Cell cultures were frozen–thawed three times and clarified by centrifugation at 1000× *g* for 5 min, and the virus supernatant was stored at −70 °C until use. The 4th passaged cells were used as the challenge virus, whereas the 11th passage ones were used for inactivated vaccine preparation. All viruses were titrated in Vero cells using a median tissue culture infective dose (TCID_50_) assay to calculate the median tissue culture infectious dose according to Reed and Muench [17].

### 2.4. Vaccine Preparation

The 11th passaged aMPV/B LN16 was inactivated with 0.1% formalin at 37 °C for 24 h as previously described [18]. To produce the vaccine, inactivated viral antigens were mixed with a novel adjuvant containing ISCOMs (Seppic, Paris, France) at 1:3 *v*/*v* or with white oil adjuvant (Exxon Mobil, Irving Texas, US) and at 1:2 *v*/*v*. The mixture was stirred well using an IKA blender (IKA, Staufen, German) at 15,600 rpm for 8 min until an emulsion was formed. Two types of inactivated vaccines, namely aMPV/B-novel and aMPV/B-oil, were prepared.

### 2.5. Experimental Design

The 21-day old SPF chickens were randomly divided into three groups: aMPV/B-novel immunized group (*n* = 38), aMPV/B-oil immunized group (*n* = 38), and unvaccinated group (*n* = 38). Priming immunization (500 μL) was administered intramuscularly to all SPF chickens in the aMPV/B-novel immunized and aMPV/B-oil immunized groups (*n* = 38 per group), and boosting immunization (*n* = 19 per group) at the same dose were administered after 3 weeks. For the unvaccinated group, the chickens were administered with phosphate buffered saline (PBS; 0.2 mL, pH 7.4) following the same procedure.

To determine the serum IgG and virus neutralizing (VN) antibody levels, blood samples were collected at 7,10, 14, 21, 28, 35, and 42 days post-priming immunization. At 7, 14, and 21 d post-priming immunization, individual spleens (*n* = 3 per group) and peripheral blood mononuclear cells (PBMCs) were also collected to determine the mRNA levels of IFN-γ and IL-4. Three weeks after priming and boosting immunization, vaccinated (*n* = 10 per group) and unvaccinated (*n* = 10) birds were challenged with 0.2 mL (10,000 TCID_50_ per chicken) aMPV LN16 strain (F4) via eye drop and intranasal routes.

### 2.6. ELISA

The serum samples were 500-fold diluted in the dilution buffer, and aMPV-specific IgG antibodies were detected using an Avian Rhinotracheitis Antibody Test Kit (IDEXX, Maine, USA) following the manufacturer’s instructions. ELISA results are presented as samples to positive control (S/P) ratios.

### 2.7. Virus Neutralization Test

Virus-neutralizing (VN) antibodies of priming or boosting immune sera were detected by a virus neutralization test as previously described [19]. Briefly, 100 μL medium containing 10^5^ Vero cells was cultured in 96-well flat-bottomed tissue-culture plates (Corning Glass Works Co., Corning, NY, USA). Then, the immune sera were heat-inactivated for 30 min at 56 °C. Next, 0.4 volume of two-fold dilution series of serum samples starting at 1:2 in DMEM were mixed with 0.4 mL of DMEM containing aMPV LN16 (F11, 2000 TCID_50_/mL) and then incubated for 1 h at 37 °C. Subsequently, 100 µL of the mixture was added to six repeated wells and incubated for 1 h. The culture medium was then changed to 1% FBS DMEM. Cytopathic effect (CPE) was recorded daily for 7 days after incubation at 37 °C in a 5% carbon dioxide atmosphere. VN titers were expressed as the reciprocal of the highest serum dilutions giving 50% reduction of plaque numbers relative to the medium controls and calculated using the method of Reed and Muench.

### 2.8. Virus and Cytokine Detection Using RT-qPCR

Total RNAs were extracted from chicken spleens at 7, 14, and 21 days post-immunization (dpi) and from choanal swabs of virus-infected chickens at 1–7 days post-challenge (dpc). RNAs were reverse transcribed to cDNA using reverse transcriptase (Vazyme, Nanjing, China) according to the manufacturer’s directions. Virus copies and mRNA expression levels of cytokine were quantified using TaqMan RT-qPCR. qPCR was performed with the primers and probes listed in Table 1. The probes were labeled with the fluorescent reporter dye FAM at the 5′-end and the quencher TAMRA at the 3′-end. Real-time RT-qPCR was performed using qRT-PCR master mix reagents (Takara, Dalian, China). The amplification and detection of specific products, including aMPV/B, interferon γ (IFN-γ), and interleukin 4 (IL-4), was performed using Applied Biosystems 7500 Fast Real-Time PCR System (Thermo Fisher Scientific, Waltham, Massachusetts, US) with the following cycle profile: one cycle at 48 °C for 30 min and 95 °C for 20 s, followed by 40 cycles of 95 °C for 3 s and 60 °C for 30 s.

### 2.9. Lymphocyte Proliferation Assay

Lymphocyte proliferation using the methyl thiazolyl tetrazolium method was assessed using a Cell Counting Kit-8 (CCK-8, Dojindo, Tokyo, Japan) according to the manufacturer’s instructions. Fresh anticoagulant blood samples (4 mL; *n* = 3 per group) were collected for the preparation of PBMCs using a chicken peripheral blood leukocyte separation fluid kit (Haoyang, Tianjin, China) following the manufacturer’s instructions. The concentration of PBMCs was determined using cell Countstar and then diluted to approximately 5 × 10^5^ to 1 × 10^6^ cells/mL. Cell suspensions (100 μL) were added to 96-well, flat-bottomed tissue-culture plates (Corning Glass Works Co., Corning, NY, USA). Then, mitogen (10 μL) containing concanamycin A (ConA; 50 μg/mL) and phytohemagglutinin (PMA; 1 μg/mL), lipopolysaccharide (LPS; 10 μg/mL), or no mitogen (control) was dispensed into each well and incubated for 72 h. LPS concentrations were 10 mg/mL of the culture medium. Lymphocyte samples cultured at each mitogen concentration were assayed in quadruplicate wells. Two hours before the termination of incubation, MST-8 (10 μL; 10 mg/mL) was added to each well, and after 4 h, absorbance was measured using an automated microtiter plate reader (Model EL310; BioTek Instruments, Inc., Winooski, VT, USA) at 450 nm. Lymphocytes were quantified using the formula: SI = (OD_450_ of stimulated cultures − machine background)/(OD_450_ of nonstimulated cultures − machine background of the negative-control group).

### 2.10. Clinical Signs

Chickens were monitored for clinical signs by a single examiner twice-daily, once in the morning and once in the afternoon at 1–7 dpc. This investigator was not aware of the chicken status (vaccinated and unvaccinated). The disease was confirmed based on the presence of nasal scab or turbid nasal fluid.

### 2.11. Histopathological Analysis

Turbinate samples from each bird were fixed by immersion in 10% neutral buffered formalin, routinely processed, and embedded in paraffin. Tissue sections were stained with hematoxylin and eosin and blindly visualized using light microscopy (Motic, Xiamen, China).

### 2.12. Immunity Duration of the Inactivated Vaccine

To determine the immunity duration induced by aMPV/B-novel, immunogenicity and protection studies were conducted up to 6 months following boosting vaccination. SPF chickens (21 days old; *n* = 60) were immunized intramuscularly with aMPV/B-novel vaccine (500 μL). After 3 weeks, they were administered with the same vaccine dose for boosting immunization. For the control group, SPF chickens (21 days old; *n* = 60) were intramuscularly injected with PBS (pH = 7.4, 0.5 mL) twice. Following boosting vaccination, blood serum samples were collected to determine the VN titers once every other month for 6 months, and the chickens were challenged with the virulent virus monthly.

### 2.13. Statistical Analysis

Statistical analysis was performed using the GraphPad software 8.0.2 (GraphPad Software Inc., San Diego, CA, USA). Two-way ANOVA with Tukey’s multiple comparison test was used for multiple comparisons. *p* < 0.05 was considered statistically significant.

## 3. Results

### 3.1. Serum IgG Antibody Levels

**Figure 1:**
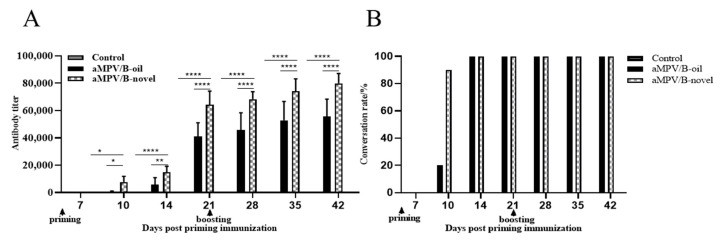
Avian metapneumovirus (aMPV) specific IgG antibody levels after priming and boosting immunization. Three-week-old chickens were immunized with two types of inactivated vaccines (0.5 mL), and the boosting immunization was administered 3 weeks later. Sera (*n* = 10 per group) were collected 1–6 weeks after prime immunization. (**A**) aMPV-specific IgG levels were determined using ELISA; (**B**) Positive rate of sera after priming and boosting immunization. aMPV/B-oil: chickens immunized with inactivated aMPV/B vaccine combined with white oil adjuvant (0.5 mL); aMPV/B-novel: chickens immunized with inactivated aMPV/B vaccine combined with a novel adjuvant (0.5 mL). Statistical significance was determined using two-way ANOVA in GraphPad Prism. **** *p* < 0.0001; ** *p* < 0.01; * *p* < 0.05.

SPF chickens (21 days old) were priming immunized (*n* = 19) and boosting immunized (*n* = 19) after 3 weeks with two inactivated vaccines, respectively. aMPV-specific antibodies from 10 chickens each group were detected at 7, 10, 14, 21, 28, 35, and 42 days post-priming immunization using an Avian Rhinotracheitis Antibody Test Kit (IDEXX, Maine, USA). As shown in Figure 1, in the aMPV/B-novel group, the serum conversion rate of aMPV-specific antibodies was 100% at 10 dpi. The mean antibody titers at 3 weeks post-priming and boosting immunization were 64,341 and 79,991, respectively. In the aMPV/B-oil group, the serum conversion rates were 10% and 100% at 10 and 14 dpi, respectively; at 3 weeks post-priming and boosting immunization, the mean antibody titers were 41,107 and 55,696, respectively. Control birds (unvaccinated) showed no increase in antibody levels. These results indicated that the aMPV-specific antibody titer in the aMPV/B-novel group was significantly higher (*p* < 0.0001) than that in the aMPV/B-oil group 3 weeks after boosting vaccination.

### 3.2. Virus Neutralizing Antibody Level

**Figure 2:**
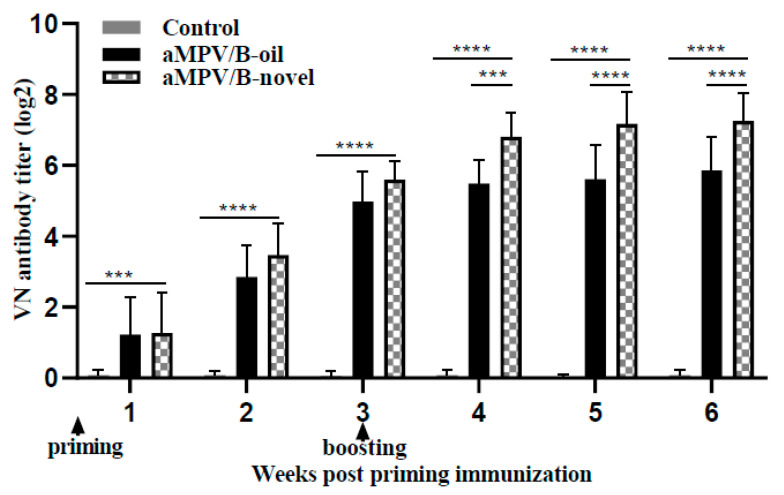
Virus neutralizing antibody levels after priming and boosting immunization. Sera neutralizing antibody titers are expressed as the reciprocal of the highest serum dilutions giving 50% reduction of plaque numbers relative to the medium controls. aMPV/B-oil: chickens immunized with inactivated aMPV/B vaccine combined with white oil (0.5 mL); aMPV/B-novel: chickens immunized with inactivated aMPV/B vaccine combined with a novel adjuvant (0.5 mL). Statistical significance was determined using two-way ANOVA in GraphPad Prism. **** *p* < 0.0001; *** *p* < 0.001.

The induction of virus neutralizing (VN) antibodies after vaccination can be a key protective immune response. Thus, we evaluated the levels of VN antibodies from 10 chickens each group after the administration of the inactivated aMPV/B vaccines. In both vaccinated groups, VN antibody were detected positive from 7 dpi, and 100% positive response was observed at 21 dpi. At 3 weeks after priming and boosting vaccination, the geometric averages of the VN antibody levels in the aMPV/B-novel group were 5.57 log2 and 7.21 log2, respectively, whereas those in the aMPV/B-oil group were 4.91 log2 and 5.79 log2 (Table 2). Thus, the VN antibody titer in the aMPV/B-novel group was 2.6-fold higher than that in the aMPV/B-oil group at 3 weeks after boosting vaccination (Figure 2). The detected VN antibodies in the unvaccinated group were negative (<2).

### 3.3. Lymphocyte Proliferation Activity in PBMCs

**Figure 3:**
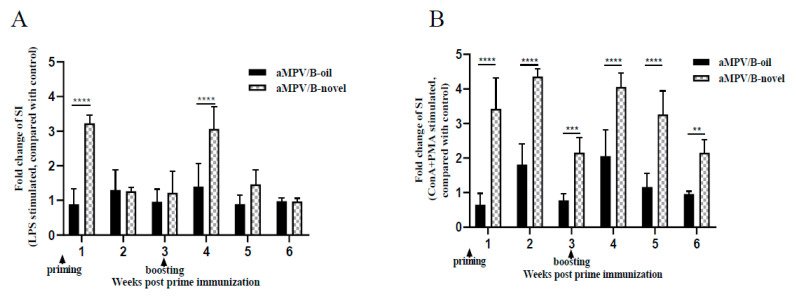
Lymphocyte proliferation in PBMCs at 1–6 weeks after priming immunization was measured after stimulation with B cell (LPS) and T cell (ConA + PMA) mitogens. SI was determined using CCK-8 kit. (**A**) PBMCs were stimulated using B cell mitogen (LPS); (**B**) PBMCs were stimulated using T cell mitogen (ConA + PMA). aMPV/B-oil: chickens immunized with inactivated aMPV/B vaccine combined with white oil adjuvant (0.5 mL); aMPV/B-novel: chickens immunized with inactivated aMPV/B vaccine combined with a novel adjuvant (0.5 mL). Statistical significance was determined using two-way ANOVA in GraphPad Prism. **** *p* < 0.0001; *** *p* < 0.001; ** *p* < 0.01.

Lymphocyte proliferation response in PBMCs can reflect the extent to which the body is affected by the vaccine. Therefore, lymphocyte proliferation in PBMCs from three chickens each group at 1–3 weeks after priming and boosting immunization with the inactivated aMPV/B vaccines was measured using the CCK-8 kit. Lymphocyte proliferation activity was presented as the stimulation index (SI) and data was presented as fold change of SI relative to control group. After stimulation with LPS, SI values of the aMPV/B-novel group were 3.2- and 3.1-fold higher than those of the control at 7 days post-priming and boosting immunization, respectively, whereas those of the aMPV/B-oil group showed no significant increase compared with the control (Figure 3A). After ConA and PMA stimulation, the aMPV/B-novel group had 2.2- to 4.4-fold higher SI values than those of the control group at 1–6 weeks post-priming immunization. The SI values of the aMPV/B-oil group showed 1.8- and 2.1-fold increases at only 2 and 4 weeks post-priming immunization (Figure 3B). These results indicated that following the stimulation by T-cell (ConA + PMA) or B-cell mitogens (LPS), the lymphocyte proliferation response was better in the aMPV/B-novel group than in the aMPV/B-oil group.

### 3.4. Th1- and Th2-Associated Cytokine Detection in Spleen

**Figure 4:**
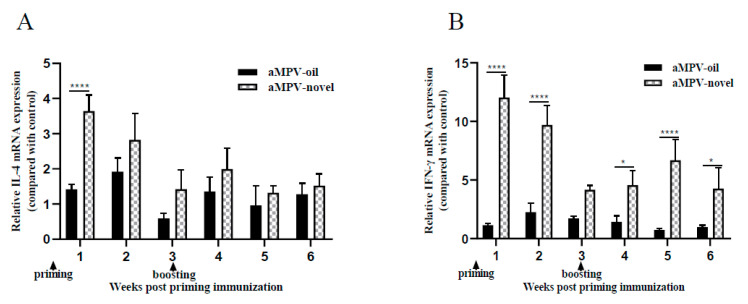
Th1 or Th2 cytokine mRNA levels were determined in spleens collected at 1–6 weeks post priming vaccination. RT-qPCR were used to determine the mRNA levels of IL-4 (**A**) and IFN-γ (**B**). Data were presented as multiples of changes in the immune group compared to the control group. aMPV/B-oil: chickens immunized with inactivated aMPV/B vaccine combined with white oil adjuvant (0.5 mL); aMPV/B-novel: chickens immunized with inactivated aMPV/B vaccine combined with a novel adjuvant (0.5 mL). Statistical significance was determined using two-way ANOVA in GraphPad Prism. **** *p* < 0.0001; * *p* < 0.05.

The mRNA expression of Th1- and Th2-associated cytokines is a sensitive marker for in vivo lymphocyte activation. Here, the mRNA levels of Th1- and Th2-associated cytokines, i.e., IFN-γ, and IL-4 cytokines in the spleen at 1–6 weeks post priming immunization with the inactivated aMPV/B vaccine were determined using qRT-PCR. Fold expression of the genes relative to control group were calculated with 28s as the reference gene. The mRNA levels of IL-4 in the aMPV/B-novel group were approximately 1.4- to 3.6-fold higher than those in the control group (Figure 4A). In addition, 4.2- to 12.0-fold increases in IFN-γ mRNA levels were observed in the aMPV/B-novel group compared with the control (Figure 4B). Meanwhile, no statistical difference in IL-4 and IFN-γ cytokine mRNA levels was observed between the aMPV/B-oil group and the control (Figure 4A,B). These results indicated that the aMPV/B-novel vaccine could promote the expression of IL-4 and IFN-γ cytokines after immunization.

### 3.5. Clinical Symptoms

**Figure 5:**
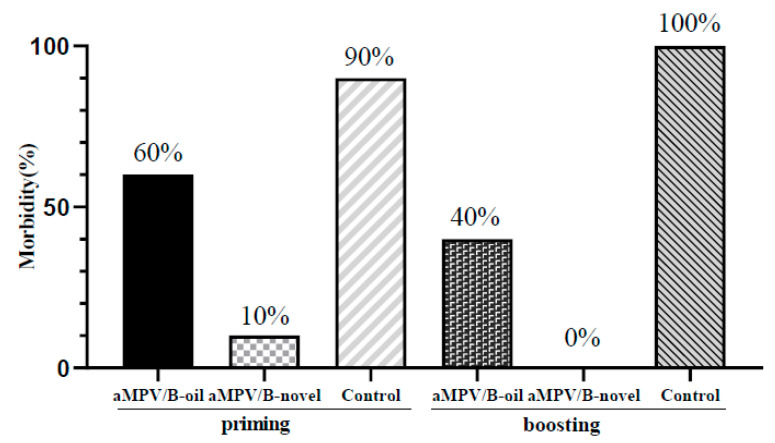
Morbidities of vaccinated and unvaccinated chickens after challenge with virulent aMPV/B. Three-week-old chickens were immunized with inactivated vaccines (0.5 mL), and the boosting immunization was administered 3 weeks later. Vaccinated and unvaccinated chickens were oculonasally challenged with 200 μL (50 μL each in each eye and nostril) aMPV/B LN16 strain (F4, 10,000 TCID_50_ per chicken). Clinical symptoms were recorded at 1–7 dpc blindly and the disease was confirmed based on the presence of nasal scab or turbid nasal fluid.

To evaluate the protective efficacy, aMPV/B-novel- and aMPV/B-oil-immunized SPF chickens were challenged with aMPV LN16 at 3 weeks after priming and boosting vaccination. Clinical signs were recorded between 1 and 7 dpc. After the virus challenge, unvaccinated chickens developed obvious clinical signs, including nasal scab and turbid nasal fluid. At 3 weeks after priming vaccination, the morbidity rates in the aMPV/B-oil, aMPV/B-novel, and unvaccinated groups were 60%, 10%, and 90%, respectively. At 3 weeks after boosting vaccination, the morbidity rates in the aMPV/B-oil, aMPV/B-novel, and unvaccinated groups were 40%, 0%, and 100%, respectively (Figure 5). These results indicated that the aMPV/B-novel provided 88.9% and 100.0% protection from clinical disease after priming and boosting immunization, whereas the protective rate of aMPV/B-oil was 33.3% and 60.0%, respectively.

### 3.6. Virus Shedding

**Figure 6:**
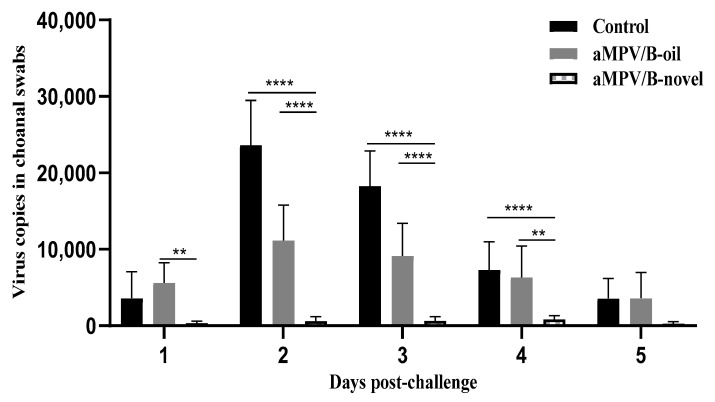
aMPV copies in choanal swabs of vaccinated and unvaccinated chickens after challenge with virulent aMPV/B. Three-week-old chickens were immunized with inactivated vaccine (0.5 mL), and the boosting immunization was administered 3 weeks later. Naive and immunized chickens were oculonasally challenged with 200 μL (50 μL each in each eye and nostril) aMPV/B LN16 strain (F4, 10,000 TCID_50_ per chicken). Choanal swabs were collected at 1–7 days after aMPV challenge and aMPV titers were determined using RT-qPCR. Control: unimmunized chickens with aMPV infection; aMPV/B-oil: chickens immunized with inactivated aMPV/B vaccine combined with white oil adjuvant (0.5 mL); aMPV/B-novel: chickens immunized with inactivated aMPV/B vaccine combined with a novel adjuvant (0.5 mL). Results are presented as mean ± SEM. Statistical significance was determined using two-way ANOVA in GraphPad Prism. **** *p* < 0.0001; ** *p* < 0.01.

To determine whether the vaccines could reduce the viral shedding of the respiratory tract, choanal cleft swabs were collected from all birds during 1–7 dpc and viral shedding was examined by RT-qPCR. As shown in Figure 6, the aMPV/B-novel vaccinated chickens exhibited 10.0-, 38.2-, 27.4-, 8.7-, and 12.7-fold lower virus copies in the choanal cleft swabs at 1–5 dpc, respectively, than the unvaccinated chickens. However, in the aMPV/B-oil group, only 1-fold lower virus copy was observed on 2 and 3 dpc, and there was no significant decline at other times. These results demonstrated that aMPV/B-novel could significantly reduce virus shedding following aMPV challenge.

### 3.7. Histopathological Analysis

**Figure 7:**
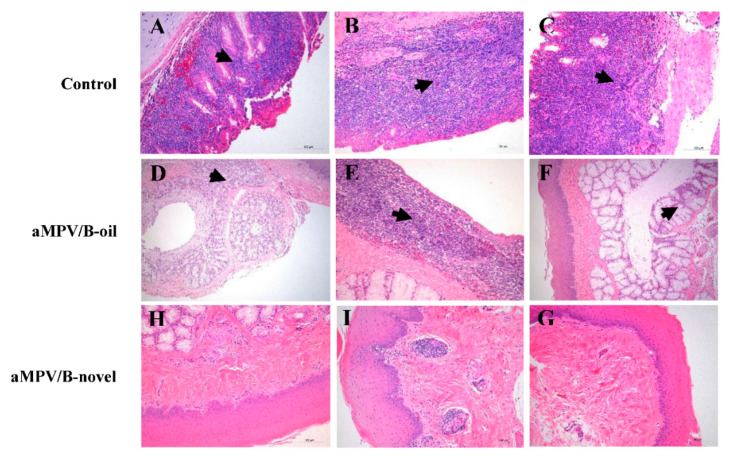
Histopathologic changes in turbinate from vaccinated and unvaccinated chickens after aMPV/B challenge. Individual turbinate tissues were collected from challenged chickens (*n* = 3 per group) at day 9 post aMPV challenge (10,000 TCID_50_ per chicken) and analyzed using histology. Nasal turbinate from unvaccinated chickens challenged with aMPV/B showing infiltration of inflammatory cells (**A**–**C**). Nasal turbinate from chickens immunized with inactivated aMPV/B-oil showing mucinous dilatation (**D**,**F**) and infiltration of inflammatory cells (**E**). No significant microscopic change was observed in the nasal turbinate from chickens immunized with inactivated aMPV/B-novel (**H**–**G**). Scale bars for H&E indicate 100 μm.

Histopathologic lesions of turbinate from three chickens in each group were observed at 9 dpc. In unvaccinated birds, exfoliation of the glandular epithelial cells and inflammatory cell infiltration were observed in the turbinate. As shown in Figure 7, in the aMPV/B-novel group, there were no obvious pathological lesions, whereas in the aMPV/B-oil group, significant inflammatory cell infiltration or mucus gland dilation was observed. These results indicated that aMPV/B-novel can prevent the development of turbinate lesions following aMPV challenge.

### 3.8. Duration of the Inactivated Vaccine

To verify the immunity conferred by aMPV/B-novel and its protection against virulent challenge, VN titers and the protective rates after challenge were determined once every other month following boosting vaccination. As shown in Table 3, all chickens still had detectable VN antibody titers to aMPV/B up to 6 months following boosting vaccination, with a mean titer of 5.79 log2. Unvaccinated chickens remained seronegative throughout the post-vaccination period. At 6 months after boosting vaccination, the protective rate of the aMPV/B-novel was still 89%. Thus, the aMPV/B-novel vaccine could provide protection against virulent challenge for at least 6 months following boosting vaccination.

## 4. Discussion

aMPV is the causative agent of acute respiratory disease in chickens and turkeys, thus causing considerable economic losses to the poultry industry. To control its spread and the prevalence of the subsequent disease in China, we developed an inactivated vaccine of aMPV/B LN16 strain and systematically evaluated its immune protection efficacy. Our results indicated that the inactivated vaccine combined with a novel adjuvant (aMPV/B-novel) could stimulate both cellular and humoral immune responses, including serum IgG and VN antibody production. In addition, boosting vaccination with the aMPV/B-novel vaccine was effective in inhibiting virus shedding and reducing turbinate inflammation. More importantly, boosting vaccination conferred 100% protection against virulent aMPV/B challenge that could last for at least 6 months. These results suggest that the inactivated aMPV/B vaccine could be used as a new vaccine candidate for aMPV/B control.

The fusion (F) protein and glycoprotein (G) are two major membrane-associated structural proteins of aMPV and play important roles in virus pathogenicity and immunogenicity [20,21]. The inactivated vaccine was developed based on the aMPV/B LN16 strain, which we have isolated previously [14]. The sequence analysis results demonstrated that the F protein of the aMPV/B LN16 strain was conserved with other aMPV isolates from different countries, thus showing at least 98.0% homology. In addition, the homology of the G protein among aMPV/B isolates ranged from 95.2% to 100%. The viral protein genes of the LN16 strain were highly similar to the only complete genome of the submitted strain VCO3/60616 (92.8–100%). These suggest that the inactivated vaccine developed in this study might match many of the circulating strains in different countries, and that it may possess protective abilities against other aMPV/B strains.

Emulsions have long been employed as vaccine adjuvants. In addition to the recruitment and activation of cells at the injection site, emulsions favor the uptake of antigen by antigen-presenting cells and their transport to the draining lymph nodes [22,23]. The activation of B cells capable of producing antigen-specific antibodies and their subsequent differentiation into long-lived memory B cells are speculated as key mechanisms by which oil-emulsion vaccines work [24]. In this study, transient increases in B cell proliferative activity were observed only at 1 and 4 weeks post vaccination in aMPV/B-novel group, which may be related to the small number of B cells in the peripheral blood [25]. However, both the aMPV/B-novel and aMPV/B-oil promoted the production of serum IgG antibodies. Particularly, the aMPV/B-novel induced a significantly enhanced virus-specific IgG antibody response compared to the aMPV/B-oil. However, high IgG serum levels are not sufficient indicators of vaccine protection; previous studies have demonstrated that intravenously administered antibodies did not protect turkeys from aMPV infection [26]. Therefore, we also conducted virus neutralization tests to validate our findings. Unlike previous studies attempting to develop vaccines but failed to detect VN antibodies [27], the average VN titer induced by our aMPV/B-novel vaccine reached 7.21 log2 at 3 weeks post-boosting vaccination, which was twice as high as that induced by aMPV/B-oil vaccine. VN antibodies are key components in the protective immune responses to viral infections because they can bind to viral particles and block them from entering host cells [28]. It has also been proved in our study that the high VN antibody levels induced by aMPV/B-novel might contribute to the protective effect conferred by the inactivated vaccine. This is because priming and boosting immunization with aMPV/B-novel provided higher protective rates of 88.9% and 100.0%, respectively, whereas with aMPV/B-oil, only 33.3% and 60.0% protection rates were achieved, respectively.

In the case of inactivated vaccines of the Paramyxoviridae family, such as Newcastle disease virus (NDV), the levels of both humoral and cellular immunity have been reported to be related to the effect of immune protection [29]. T cells are necessary for the clearance of NDV, whereas virus-neutralizing antibodies are essential to protect against the clinical signs but cannot prevent viral shedding [30]. To investigate whether our inactivated vaccine activated cellular immunity, we evaluated the lymphocyte proliferation activity of PBMCs and found a significant increase in T cell proliferation in the aMPV/B-novel group, but not in the aMPV/B-oil group. Consistently, in comparison with the unvaccinated and aMPV/B-oil-immunized chickens, the aMPV/B-novel vaccinated chickens exhibited 8.7- to 38.2-fold lower virus copies in their choanal cleft swabs at 1–5 dpc. These results suggest that T cells are also necessary for the clearance of aMPV.

IFN-γ produced mainly by T and NK cells is a typical Th1 cytokine that can induce the differentiation of T and B cells. It can also promote the proliferation of B cells [31]. IL-4 is a representative Th2 cytokine that can promote the differentiation of T and B cells and expedite the production of IgM, IgG, and IgA in humoral immunity [32]. After priming or boosting immunization with the aMPV/B-novel, increases in both IFN-γ and IL-4 mRNA levels were detected in the spleen, consistent with the proliferative activity of lymphocytes. However, the rise in IFN-γ mRNA levels was higher than that in IL-4, which might promote Th1/Th2 balance drifting to the Th1 population. The Th1-biased paradigm has been previously shown to play an important role in inhibiting the inflammatory response and preventing airway injury [33]. Furthermore, IFN-γ decreases morbidity, mortality, and viral shedding induced by virulent viruses, such as NDV and avian influenza H5N9 [34,35]. This might explain why no inflammatory cell infiltration in the turbinate nor obvious clinical symptoms were observed after boosting vaccination with the inactivated aMPV/B-novel vaccine.

## 5. Conclusions

Based on the aMPV/B LN16 strain, we developed an inactivated aMPV/B vaccine and systematically evaluated the immune response after vaccination and the vaccine protection after viral challenge. The inactivated aMPV/B-novel vaccine activated both humoral and cellular immunity. Boosting vaccination with the inactivated aMPV/B-novel vaccine provided 100% protection against aMPV/B infection with exhibited reduced virus shedding and suppressed turbinate inflammation. Besides, the protective immunity could last for at least 6 months. These results suggest aMPV/B-novel as a new vaccine candidate. The successful development of the first aMPV/B vaccine in China will contribute to aMPV/B control and provide a foundation for disease prevention in the poultry industry.

## Figures and Tables

**Table 1:** Real-time quantitative RT-PCR probes and primers.

RNA Target	Probe/Primer Sequence (5′-3′)
aMPV/B	Probe CTGGTGTTATCAGCCTTAGGCTTGACGCT
	F AATAGTCCTCAAGCAAGTCCTCAGA
	R CTGTTGTAATTTGACCTGTTCTA CACT
28s	Probe GCATGGCTTAATCTTTGAGACAA
	F ATCCTGCCAGTAGCATATG
	R GCCGTGCGTACTTACACGT
IL-4	Probe CTCTTCCTCAACATGCGTCAGCTCCTGAATGCCAG
	F TGCCACAAGAACCTGCAGGG
	R AAGTAGTGTTGCCTGCTGCC
IFN-γ	Probe TGCCCTGCGTCAGGACTCTAA
	F ATCTCGAAAAACAACCTTCCTGATGGC
	R TTCCAGCCCAAGTGGAGCCGG

**Table 2:** Virus neutralizing antibody levels after priming and boosting immunization.

Group	Geometric Mean VN Titers (log2) ± Standard Error at Weeks Post Immunization					
	1	2	3	4	5	6
aMPV/B-oil ^a^	1.27 ± 0.91	2.72 ± 0.85	4.91 ± 0.82	5.45 ± 0.62	5.54 ± 0.90	5.79 ± 0.89
aMPV/B-novel ^b^	1.30 ± 0.98	3.35 ± 0.84	5.57 ± 0.50	6.78 ± 0.64	7.12 ± 0.87	7.21 ± 0.76

^a^ Chickens immunized with inactivated aMPV/B vaccine combined with white oil (0.5 mL); ^b^ chickens immunized with inactivated aMPV/B vaccine combined with a novel adjuvant (0.5 mL).

**Table 3:** Immunity duration of the inactivated aMPV/B-novel vaccine.

		Time after Boosting Vaccination/Months					
		1	2	3	4	5	6
VN titer	Vaccinated ^a^	7.09 ± 0.92 ^c^	6.59 ± 0.51	6.17 ± 1.17	5.57 ± 0.79	5.04 ± 1.39	5.28 ± 1.34
	Control ^b^	<2	<2	<2	<2	<2	<2
Morbidity	Vaccinated	0%	0%	10%	0%	10%	10%
	Control	90%	100%	100%	90%	100%	90%
Protective rate		100%	100%	90%	100%	90%	89%

^a^ Chickens immunized with inactivated aMPV/B vaccine combined with a novel adjuvant (0.5 mL). ^b^ Chickens intramuscularly injected with PBS (pH = 7.4, 0.5 mL). ^c^ Geometric mean VN titers (log2) ± Standard error.
